# Physical-Exercise-Induced Antioxidant Effects on the Brain and Skeletal Muscle

**DOI:** 10.3390/antiox11050826

**Published:** 2022-04-23

**Authors:** Jennyffer Souza, Rodrigo Augusto da Silva, Débora da Luz Scheffer, Rafael Penteado, Alexandre Solano, Leonardo Barros, Henning Budde, Andrés Trostchansky, Alexandra Latini

**Affiliations:** 1 Department of Biochemistry, Laboratory of Bioenergetics and Oxidative Stress—LABOX, Federal University of Santa Catarina, Florianópolis 88037-100, Brazil; jennyffer_souzaa@hotmail.com (J.S.); dschefferlabox@gmail.com (D.d.L.S.); alexandre_solano@hotmail.com (A.S.); lbarroslabox@gmail.com (L.B.); 2 Epigenetic Study Center and Gene Regulation—CEEpiRG, Program in Environmental and Experimental Pathology, Paulista University—UNIP, São Paulo 05508-070, Brazil; dasilva.rodrigo.a@gmail.com; 3 Department of Anatomy, Institute of Biomedical Sciences, University of São Paulo, São Paulo 05508-070, Brazil; 4 Human Performance Research Group, Center for Health and Sport Sciences, Universidade do Estado de Santa Catarina, Florianópolis 88037-100, Brazil; penteado.r@hotmail.com; 5 Institute for Systems Medicine, Faculty of Human Sciences, MSH Medical School Hamburg, 20457 Hamburg, Germany; henning.budde@medicalschool-hamburg.de; 6 Facultad de Medicina, Universidad de la Republica, Montevideo 11800, Uruguay; trocha@fmed.edu.uy

**Keywords:** NRF2, oxidative stress, exercise, brain, tetrahydrobiopterin, neopterin, epigenetics

## Abstract

Erythroid-related nuclear factor 2 (NRF2) and the antioxidant-responsive-elements (ARE) signaling pathway are the master regulators of cell antioxidant defenses, playing a key role in maintaining cellular homeostasis, a scenario in which proper mitochondrial function is essential. Increasing evidence indicates that the regular practice of physical exercise increases cellular antioxidant defenses by activating NRF2 signaling. This manuscript reviewed classic and ongoing research on the beneficial effects of exercise on the antioxidant system in both the brain and skeletal muscle.

## 1. Introduction

There is a large body of evidence demonstrating the central role of NRF2 (erythroid-related nuclear factor 2) activation in the beneficial effects induced by the regular practice of moderate physical exercise [1,2,3]. NRF2 is a transcription factor that is activated under electrophilic stress and translocated from the cytosol to the nucleus where it interacts with antioxidant-responsive elements (AREs) to promote cellular protective responses [4]. Evidence also suggests that NRF2 can be activated by phosphorylation [5,6,7] by specific kinases, contributing to the global enhancement of more than 250 genes, mainly to induce an antioxidant and detoxifying environment, anti-inflammatory responses, proteasomal and autophagic degradation, mitochondrial activity, and therefore metabolism. The NRF2/ARE-mediated response will finally elicit an anti-inflammatory status, favoring mitochondrial energy production, mitochondrial dynamics, autophagy, DNA repair, cell proliferation, thereby contributing to increased cell survival [8,9]. The cytoprotective pathways activated by the nuclear translocation of NRF2 are known to counterbalance toxic processes, including mitochondrial dysfunction, oxidative stress and neuroinflammation, which are considered the pathophysiological bases of many neurodegenerative diseases. In this scenario, the enhancement of the NRF2/ARE pathway has been proposed as a promising therapeutic avenue for these diseases [10,11,12,13,14].

Physical exercise has emerged in recent decades as a non-pharmacological tool to induce neuroprotection; for example, there are several reports showing the improvement of motor and non-motor symptoms of individuals affected by Parkinson’s disease [15], the protection of dopaminergic neurons from toxicity [16], the prevention of toxic protein accumulation in Alzheimer’s disease, and others [17]. However, the molecular mechanisms involved in the beneficial effects of physical exercise on the CNS are not fully understood. The objective of the present work was to review how exercise modulates the expression, content and downstream signaling of NRF2 by activating the synthesis of tetrahydrobiopterin (BH4) and modulating epigenetic profiles. Our group has demonstrated that intermediates of BH4 metabolism are direct activators of NRF2, promoting increased cellular redox activity. In addition, the cytoprotective environment induced by exercise-linked NRF2 activation will stimulate permissive gene expression, contributing to enhanced transcription activity of cytoprotective genes, including NRF2 itself. DNA methylation is one of the most understood epigenetic mechanisms that cells use to control gene expression, and the hypermethylation of specific regions of the genome is believed to explain how exercise regimes can sustain its pleiotropic effects. Finally, as has been addressed in the literature, BH4 metabolism contributes to DNA methylation by actively interacting with one-carbon metabolism.

## 2. The Antioxidant System

The cellular antioxidant response is finely regulated at the transcriptional level by the master transcription factor NRF2 [18]. NRF2 mainly regulates the gene expression of cytoprotective phase II detoxification and antioxidant enzymes through a promoter sequence known as ARE [4]. The NRF2/ARE system regulates the transcription of approximately 250 genes, including antioxidants that are crucial for cellular redox control in the brain and skeletal muscle. Some of these genes encode for the antioxidant enzymes superoxide dismutase (SOD), glutathione peroxidase (GPx), glutathione reductase (GR), hemeoxygenase (HO-1), peroxiredoxin, thioredoxin reductase, thioredoxin, and metallothionein. The increase in the protein content of these enzymes has been correlated with several beneficial effects on the brain and skeletal muscle (Table 1).

Under basal conditions, NRF2 is bound to KEAP1 in the cytosol, which negatively regulates its transcriptional activity by specifically binding to its amino-terminal regulatory domain, thereby stimulating its rapid degradation by ubiquitination [43]. This reaction is dependent on the 26S proteasome and keeps the cellular NRF2 at very low concentrations in the cytosol [43]. It is the association between KEAP1 and the actin cytoskeleton that prevents this complex from entering the nucleus, limiting the basal activity of NRF2 [43]. KEAP1 contains two NRF2-protein-interaction domains, the BTB domain (bric-a-brac, tramtrack, broad complex) in the N-terminal region, and the Kelch domain or DGR (double glycine repeat domain) in the C-region terminal. The BTB domain is responsible for the homodimerization of KEAP1, which inhibits NRF2. On the other hand, NRF2 can bind to DGR through two types of bonds in the Neh2 domain, one of high affinity and the other of low affinity. The high-affinity site allows NRF2 to bind to KEAP1, but the low-affinity site prevents the movement of NRF2 and positions lysine residues within the Neh2 region for ubiquitination. KEAP1 facilitates the Cul3-mediated poly-ubiquitination of NRF2 leading to its proteasomal degradation [44]. Under oxidative stress, the KEAP1/NRF2 association is impaired by electrophilic activity that antagonizes the action of KEAP1 [43], provoking NRF2 translocation to the nucleus, and consequently the induction of ARE-mediated transcription of antioxidant elements [45].

NRF2 stabilization is crucial for the cellular antioxidant system to be activated. KEAP1 is rich in cysteine residues and is the target of oxidants, which can covalently modify these residues. Four KEAP1 cysteines (C257, C273, C288, and C297) are particularly reactive and located in the intermediate region of the protein [46]. Modifications to these cysteines alter the conformation of KEAP1, leading to the dissociation and nuclear translocation of NRF2. In addition, a modification of C151 located in the N-terminal BTB domain is necessary to stabilize NRF2, possibly inducing a conformational change that favors the accessibility of C273 and C288 to the cytoplasm [47]. When NRF2 translocates to the nucleus, it associates with the small musculoaponeurotic fibrosarcoma (Maf) protein, a family of AP-1 transcription factors, which have the Maf-recognition element (MARE) that resembles the central region of the nucleus ARE [48]. NRF2 forms a heterodimer with Maf to bind to the ARE, NRF2 recognizes the ARE nucleus and Maf binds to the ARE 3′ end dinucleotide G (guanine) C (cytosine) [49], regulating the transcription of antioxidant genes during oxidative stress [45].

NRF2 phosphorylation has been demonstrated to occur in serine 40 by PKC, inhibiting the action of KEAP1 in response to phorbol 12-myristate 13-acetate, which is an antioxidant-response inducer [6]. NRF2 phosphorylation results in the translocation of the protein to the nucleus and the consequent activation of ARE-mediated transcription [5,6]. This signaling has been proven to occur by using PKC inhibitors when NRF2 translocation was inhibited [5,6]. In addition to PKC, PI3K can also phosphorylate NRF2 and be associated with the regulation of ARE, as it favors the translocation of NRF2 to the nucleus and the induction of transcription of antioxidant enzymes [7]. Although the nuclear translocation of NRF2 and the consequent induction of the transcription of antioxidant enzymes has been shown to be predominantly cytoprotective, there is some evidence that excessive activation of NRF2 can exert dual effects in certain tissues, such as the heart muscle. Pre-clinical studies have shown that the overactivation of NRF2 becomes pathological, causing reductive stress [50] on the heart in conditions in which myocardial autophagy is impaired, such as diabetic, hypertensive and ischemic cardiomyopathies, thereby compromising the adaptation of cardiac function under hemodynamic stress [51,52]. The mechanism behind this double effect of NRF2 is still not very well defined, and studies of this harmful effect on the brain and skeletal muscle are scarce. To date, there is no evidence that physical exercise may enhance this detrimental facet of the NRF2/ARE pathway, in the heart, skeletal muscle or the brain. However, this faceted activity of NRF2 broadens the view of the importance of the appropriate cellular redox homeostasis, which is an equilibrium between oxidative and reductive stresses, on biological functions.

### 2.1. NRF2 and Central Nervous System

The NRF2 system is widely expressed in the CNS, and it is modulated in response to both acute cerebral insults and chronic neurodegenerative diseases. Importantly, NRF2 is an important regulator of inflammation in the brain. The dysregulation of these mechanisms has been suggested to contribute to brain injury.

Animal studies have shown that the genetic suppression of the NRF2 system can exert increased neurotoxicity with widespread astrogliosis [53]. A predominant pro-inflammatory microglial phenotype was reported in dopamine-metabolizing brain areas (striatum and ventral midbrain) of NRF2-deficient animals. Indeed, changes in the expression of pro-inflammatory markers such as cyclooxygenase-2 (COX-2), inducible nitric-oxide synthases (iNOS), IL-6, and tumor necrosis factor-α (TNF-α) were observed in addition to lower contents of anti-inflammatory markers [54]. These changes were mainly observed in areas with high levels of oxidative stress, supporting the protective role of NRF2 [55].

The cytoprotective effectiveness of the NRF2 system declines with age, and aging is a strong risk factor for the development of neurological diseases. As can be expected, increased oxidative stress and severe neuroinflammation occurs in the aging brain. However, it is not known whether this impairment is linked to deficient NRF2 transcription machinery, since NRF2 is transported to the nucleus [56].

### 2.2. NRF2 Activation by Physical Exercise

**Figure 1:**
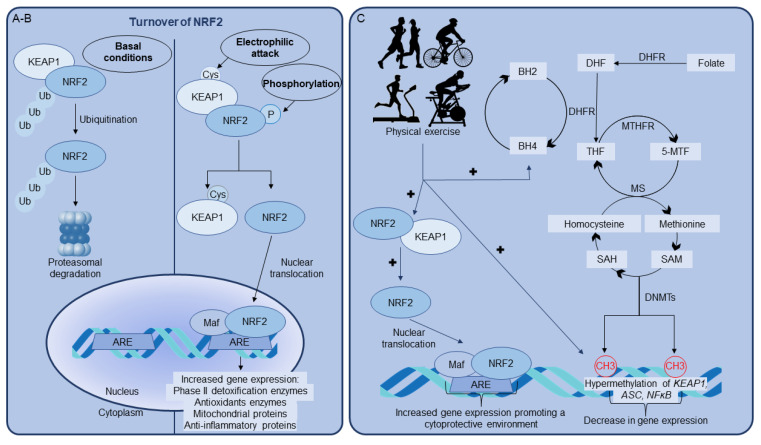
Regulation of the erythroid-related-nuclear-factor-2 (NRF2) pathway mediated by physical exercise. (**A**) Under basal conditions, cytosolic NRF2 is maintained at low levels by ubiquitin-mediated proteasomal degradation. (**B**) Electrophilic stress and NRF2 phosphorylation can induce NRF2 nuclear translocation and further interaction of the transcription factor with the antioxidant-responsive element (ARE). The interaction with ARE mediates the transcriptional activation of many genes encoding phase II drug-metabolizing and antioxidant enzymes, or proteins that will enhance mitochondrial activity and number, and promote an anti-inflammatory status. (**C**) Signaling pathways activated by physical exercise. The regular practice of physical exercise positively regulates NRF2 nuclear translocation, the synthesis of tetrahydrobiopterin (BH4) and epigenetic modifications, including DNA methylation. DNA methylation could be the result of an interplay among methionine, folate and BH4 pathways. Folate is transformed into tetrahydrofolate (THF) by the enzyme dihydrofolate reductase (DHFR), the same enzyme that catalyzes the reduction of dihydrobiopterin (BH2) into BH4 in the BH4 biosynthetic pathway. THF is transformed to 5-methyltetrahydrofolate (5-MTF) by MTHFR and converted back to THF by methionine synthase (MS), allowing the methylation of homocysteine to methionine. The latter is then transformed into S-adenoslymethionine (SAM), which can donate a methyl group for DNA methylation, leading to the formation of S-adenosylhomocysteine (SAH) and methylated DNA. The enzymes involved in DNA methylation are DNA methyltransferases (DNMTs), which transfer the methyl group from SAM to DNA, leading to methylation of the promoter region of *KEAP1* gene, decreasing its expression and favoring NRF2 translocation. In addition, the hypermethylation of the promoter region of the *ASC* gene and *NFkB*, which encode proteins involved in promoting an anti-inflammatory status, will promote an anti-inflammatory environment.

The activation of NRF2 can also occur in response to the transient stress induced by physical exercise. Growing evidence has shown the positive effects of acute and chronic physical exercise on the redox system and their beneficial effects on health (Figure 1). Several studies have proposed that both resistance and endurance exercise can lead to a perturbation of cellular redox homeostasis by increasing reactive species formation [57,58,59]. Although exercise has been shown to activate NRF2, many differences are described according to the practitioner’s age and the type, intensity, and duration of the training exercise.

Acute aerobic exercise of moderate intensity (i.e., 30 min cycling) significantly increased NRF2 content in peripheral blood mononuclear cells in young and older men [60]. However, the enhancement of NRF2 nuclear translocation was only observed in the immune cells of the younger group. The nuclear localization of NRF2 occurred with increased content of downstream-related proteins, including HO-1 and NQO1. These data suggest that aging impairs the antioxidant defense of the body, but that it can be partially stimulated by the practice of physical exercise [60]. Additionally, when the same group of young men was subjected to acute aerobic exercise at high intensity, another group of antioxidant proteins was also up-regulated. The exercise protocol elicited greater activity of GR, indicating that the degree of NRF2 activation also depends on the intensity of the aerobic exercise [61]. Although NRF2 nuclear translocation is reduced in older men [60], a pre-clinical study in C57BL6 aged male mice (23 months old) showed that this impairment can be reversed if the exercise session is repeated (chronic exercise) [62]. After an acute treadmill-endurance exercise, old mice were highly susceptible to oxidative stress, but after moderate chronic physical training (six weeks) there was an increase in the adaptive redox homeostasis, promoting increased NRF2 activation and protecting the heart from oxidative stress [62].

Differences in the exercise-time-dependent activation of NRF2 were also demonstrated in a study with C57BL6 young mice (two months old) submitted to 1 h and 6 h treadmill running. After the 6 h run, there was a significant increase in the activation of the KEAP1/NRF2/ARE pathway in skeletal muscle, but this increase was not observed after a 1 h run, indicating that the longer the exercise duration the higher the cellular antioxidant response [63].

The regular practice of exercise has also been associated with beneficial effects on the central nervous system. A recent study showed that vigorous and longer-duration aerobic exercise increased the content of NRF2 in the hippocampus and of HO-1 in the cortex after treadmill-exercise training [58]. Additionally, rats undergoing regular treadmill exercise for four weeks showed a protective effect against oxidative stress on dopaminergic neurons, by inducing the expression of NRF2, in a parkinsonism model [16]. Similar results were observed in the same animal model when rodents were submitted to a six-week-treadmill-exercise program, where the NRF2/ARE pathway was enhanced in the nigrostriatal pathway, generating a protective effect against the development of hemiparkinsonism [64]. The data presented are in agreement with the hippocampal down-regulation of the NRF2/ARE signaling pathway during neuroinflammation, chronic oxidative stress, and cognitive impairment [65].

Although NFR2 activation has been traditionally associated with enhanced intracellular cytoprotection, which is characterized by an increased resistance to oxidative stress, inflammation, and increasing capacity to generate energy, it has also been demonstrated that the persistent activation of the system is deleterious, provoking oxidative stress and the impairment of signaling functions [66]. This phenomenon has been described as reductive stress and elicits a condition in which ROS levels are below their physiological levels, perturbing the cell’s signaling functions [67]. Indeed, adipocyte differentiation [68], the activation of UCP-1 (uncoupling protein 1) [69], and cardiac remodeling [50] are some of the many examples where ROS are essential for signaling physiological processes [70]. A persistent reductive stress is as harmful as oxidative stress and is implicated in many pathological processes [67]. It has been shown that reductive stress blocks cell differentiation [71] or results in cancer, diabetes, or cardiomyopathy [72].

## 3. Role of BH4 on NRF2/ARE Pathway Activated by Physical Exercise

BH4 is a pteridine that acts as a mandatory cofactor for the activity of phenylalanine, tyrosine, and tryptophan hydroxylases, for alkylglycerol monooxygenase, and all isoforms of nitric-oxide synthases (NOS) [73]. Therefore, BH4 is essential for the biosynthesis of the neurotransmitters, dopamine and serotonin, for the catabolism of phenylalanine and ether lipids, and for the formation of nitric oxide.

Three biosynthetic pathways are responsible for tuning the intracellular concentrations of BH4: the de novo, the salvage, and the recycling pathways. The de novo pathway synthesizes BH4 from guanosine triphosphate through the sequential action of guanosine triphosphate cyclohydrolase I (GTPCH), 6-pyruvoyl tetrahydropterin synthase (PTPS), and sepiapterin reductase (SPR) [73]. GTPCH is the rate-limiting enzyme of the de novo pathway and is transcriptionally regulated by inflammatory mediators, including interferon-γ (IFN-γ), lipopolysaccharide (LPS), interleukin-1β (IL-1β), and hydrogen peroxide [74]. Thus, under inflammatory conditions, the expression of *GCH1*, the gene that encodes for GTPCH, is up-regulated several times; however, since the other enzymes in the pathway are not inducible by inflammation, a metabolic pseudo-blockage is generated, resulting in the production of neopterin [75]. Indeed, neopterin has been used as a sensitive marker of immune-system activation for several decades [76]. The salvage pathway synthesizes BH4 by utilizing intermediates from the de novo pathway to form sepiapterin, which is later converted to BH4 by the action of the enzymes SPR and dihydrofolate reductase (DHFR) [74]. The recycling pathway is a mechanism that maintains adequate intracellular concentrations of BH4 without the need for energy expenditure in tissues with a high demand for this pteridine, i.e., in the liver for the proper metabolism of phenylalanine. After BH4 is used as an essential cofactor, the molecule is oxidized to quinonoid dihydrobiopterin (qBH2) and recycled back to BH4 by the action of dihydropteridin reductase (DHPR) [73].

BH4 is traditionally known due to its activity as an enzyme cofactor [73]. However, our group and others have shown that the BH4 pathway is essential for maintaining the activity of mitochondria and the antioxidant system, and for inducing an anti-inflammatory scenario [77,78,79]. This has positioned BH4 metabolism as a potential new target to prevent or attenuate the cytotoxicity linked to chronic inflammatory diseases. In this context, our lab has shown that a single intracerebroventricular administration of neopterin (a dose that will slightly increase the levels of the compound in the cerebrospinal fluid) to naïve mice provoked the increase of the antioxidant response by augmenting glutathione levels and the activity of GPx, which are downstream components of the NRF2/ARE-pathway activation, in the brain [80]. In addition, the treatment also prevented the brain’s massive increase of pro-inflammatory cytokines after an intraperitoneal LPS challenge, suggesting that neopterin also maintains the balance between NRF2 and the master regulator of inflammation, nuclear factor-κB (NF-kB) [81].

To try to dissect the mechanisms involved in the antioxidant effect of neopterin, our group also exposed nerve cells obtained from mammals, humans, and rodents to neopterin. We observed that the pre-conditioning with neopterin prevented the activation of the inflammasome, which is a macromolecular protein complex that mediates the synthesis of IL1-β through the activation of pro-inflammatory caspase [82], and also the production of reactive oxygen species (ROS) induced by LPS and IFN-γ [81]. The treatment with neopterin to naïve cells provoked the rapid nuclear translocation of NRF2, the production of HO-1, and increased mitochondrial activity [76,81]. The latter was evidenced by increased activity of complexes I and IV and by increased basal respiration. The enhanced mitochondrial activity was accompanied by reduced lactate formation, indicating that neopterin increased mitochondrial oxidative metabolism and reduced anaerobic glycolysis. Furthermore, our group also observed that neopterin exposure provoked the formation of very low concentrations of superoxide radicals, which can be responsible for an electrophilic attack and consequent activation of the NRF2/ARE pathway [81].

The antioxidant, anti-inflammatory, and mitochondrial-activator properties shown by neopterin might also be responsible for the mnemonic effects of the molecule. Our group demonstrated that neopterin enhances aversive memory acquisition by reducing the threshold to generate hippocampal long-term potentiation, which is an essential mechanism for memory formation [83].

It is widely described that all the above-mentioned cytoprotective mechanisms are induced by the regular practice of moderate-intensity physical exercise. These effects have been described in the blood, muscle, liver, and brain of animals and also in the blood and urine of humans [58,64]. Recently, we have shown that moderate running exercise increases urinary neopterin levels under basal conditions and prevents exacerbated immune-system activation under an inflammatory scenario [84]. Other groups have also confirmed a positive correlation between physical exercise and the increase in BH4 and neopterin in human biological fluids, as shown in Table 2.

Since we have characterized the cytoprotective effects of the BH4 metabolic pathway on the brain of experimental systems and cultured cells, it is feasible that part of the effects induced by exercise might be mediated by the activation of BH4 metabolism. The relationship between BH4 metabolism and NRF2 activation remains unclear, but in vitro studies using *Gch1*-deficient macrophages indicated the existence of a NRF2/*GCH1*/BH4 axis, which has the function of protecting against oxidative stress, with *GCH1* being one effector switch [77,96]. Furthermore, our group demonstrated that neopterin can activate the expression, content, and activity of NRF2 in vitro [81], and increase the content of the downstream proteins of the pathway [81]. Independently, it has been also shown in an in vitro study with macrophages that NRF2 requires BH4 for its activation [96]. The correlation between neopterin and the beneficial effects of physical exercise has led sport and exercise medicine to use it as an indicator of immune-system activation. Its use as a biomarker is also growing when compared with other traditional inflammatory markers [97,98].

Regarding plasma levels of BH4 increase, it has been shown that the levels can increase rapidly and can be sustained for up to 2 h after the practice of strong physical exercise in young and middle-aged individuals, pointing to a temporal response of this metabolic pathway with exercise intensity [85,86].

During physical exercise, there is an inherent consumption of energy, generation of ROS, and consequent activation of the immune system [99]. Increased plasma levels of neopterin have been demonstrated after running [87], ergometer [88,89], and even after ultra-endurance competition [90]. This increase has also been reported in urine after running [91], rugby [92], bodybuilding competition [93], triathlon [94] and ultra-marathon [95]. The rapid and transient increase in BH4 and neopterin after exhausting exercises can be also interpreted as the result of an oxidative burst followed by the activation of monocytes and macrophages, reflecting the immune activation stimulated in this context [88]. The presented scenario indicates that BH4 metabolism can behave as a biomarker of inflammation induced by high-intensity physical exercise, but also as a cytoprotective and neurological mediator of the beneficial effect generated by physical exercise on the antioxidant system, including the activation of NRF2 [3]. 

## 4. Epigenetics as a Key Player in NRF2 Upregulation Induced by Physical Exercise

The term epigenetics was conceived by Conrad Waddington in 1940 to describe the possible causal processes acting on genes that regulate phenotype [100]. Over the years, the definition and concept of epigenetics have gradually evolved to mean the existence of a process that alters gene activity without changing the nucleotide sequences [101]. Epigenetic profiles are controlled by several biochemical processes, including DNA methylation, histone modification, and non-coding-RNA-modulated expression. These mechanisms mainly control gene expression at the transcriptional level through chromatin compaction and/or relaxation, thereby blocking/allowing the accessibility of transcription factors to the promoter region [102]. Epigenetic processes can also prevent protein translation by inactivating or degrading messenger RNA (mRNA) through the action of interfering microRNAs (miRNA) [103,104].

### 4.1. DNA Methylation

DNA methylation is the most characterized epigenetic alteration and consists of the covalent addition of a methyl group catalyzed by DNA methyltransferases (DNMTs). DNMTs transfer a methyl group from S-adenosylmethionine (SAM) to the 5′ carbon of a cytosine that usually precedes guanine (CpG dinucleotide), forming 5-methyl cytosine (5-meC). DNA methylation of CpG regions, called CpG islands, is usually associated with the inhibition of gene expression [105]. DNA methylation can be also modified by a family of 2-oxoglutarate- and Fe (II)-dependent dioxygenase enzymes named TET translocation proteins (TET-eleven-translocation). These proteins, TET1–3, can oxidize 5-meC into 5-hydroxymethylcytosine (5-hmC) and 5-carboxycytosine (5-caC). The decarboxylation of 5caC will provoke the demethylation of the DNA [106]. Studies have suggested a direct action of CpG-island hypermethylation in the regulation of *NRF2* transcriptional activity [107]. The downregulation of *NRF2* by DNA methylation has been described in a cellular model of Alzheimer’s disease [108], diabetic cardiomyopathy [109], and especially in different types of cancers [107]. Furthermore, DNA methylation has been associated with the protective effect of physical exercise [110]. Although exercise-induced redox disturbances can act as downstream modulators of the epigenetic machinery, data demonstrating a direct exercise-induced epigenetic modulation of *NRF2* gene expression are scarce [111]. The increased activation of NRF2 has been attributed to the hypermethylation of *KEAP1*, favoring NRF2translocation to the cell nucleus [112,113]. In agreement, it has been shown that running exercise can reverse *NRF2* promoter hypermethylation in a pre-clinical osteoporosis model, thereby attenuating the suppression of antioxidant enzymes [114]. In addition, it is well established that physical exercise increases ROS production, and recent studies indicate that ROS can activate TET DNA demethylases and cause hypomethylation of the NFE2L2 promoter, resulting in NRF2 activation [115,116].

### 4.2. Histone Modifications

Histone modification is another key mechanism in the regulation of gene expression. An octamer of histone proteins makes up the main repeating element of chromatin, the nucleosome. Histones have N-terminal tails that are prone to a variety of post-translational changes, with histones H3 and H4 being the most studied concerning gene-expression regulation [102,117,118]. These modifications are controlled by four groups of enzymes: histone acetyltransferases (HATs), histone methyltransferases (HMTs), histone deacetylases (HDACs), and histone demethylases [119,120]. In this scenario, it has been demonstrated that increased histone acetylation occurred in the hippocampus of rats that were subjected to physical exercise. This epigenetic modification was associated with improved neurocognition and aversive-memory performance [121,122].

The HDACs family is composed of sirtuins (Sirts), which due to their NAD+-dependence on the deacetylase activity, can regulate redox reactions by modulating transcription factors that control the expression of antioxidant enzymes [123]. NRF2 has been suggested to be a downstream regulator of Sirt1 in a cardiac-ischemia model [124]. On the other hand, Sirt2 has been associated with the deacetylation of *NRF2* and consequent reduction of its total cellular and nuclear levels, leading to a decrease in its transcriptional activity [125]. NRF2 levels can also be modulated by Sirt2 through Akt phosphorylation, leading to the regulation of glutathione concentrations, suggesting a role in the NRF2/ARE system [126]. Although studies demonstrating the association between physical exercise, Sirts and NRF2 are scarce, the available evidence that exercise modulates sirtuins [127,128,129] and that they can act in the regulation of NRF2 [124,125,126] reveal an area to be studied and a possible mechanism generated by the practice of physical exercise.

### 4.3. Post-Transcriptional Regulation

Recently, non-coding RNAs (ncRNAs), especially long non-coding RNAs (lncRNAs), have been implicated as important epigenetic modulators due to the ability to neutralize miRNAs by their sponge activity. LncRNAs are also capable of directing DNA methylation and histone modifications, thereby modulating gene expression [130]. NcRNAs can act as competitive endogenous RNAs to absorb and suppress the activity of bound miRNAs, effectively derepressing other targets of these miRNAs [131]. The regulation of gene expression by lncRNAs at the epigenetic, transcriptional and post-transcriptional levels have been widely studied, and there are strong indications that the expression of certain lncRNAs can modulate the effects of physical exercise (Table 3).

LncRNA studies provide new insights into the regulation of beneficial exercise-induced effects, but despite NRF2 having a central role in these effects, studies demonstrating the involvement of lncRNA in the regulation of exercise-induced *NRF2* expression are scarce. Following aerobic exercise, miR-340-5p has been shown to play a role in the post-transcriptional regulation of NRF2 expression in mouse skeletal muscles [141].

## 5. Effects of BH4 on Epigenetic Modulation Induced by Physical Exercise

Folate and BH4 are chemically defined as pterins due to the presence of the heterocycle ring pteridine. Different from BH4, folate is an essential vitamin that needs to be included in the diet in order to modulate metabolism as a micronutrient. Dietary folate requires the activity of DHFR to be converted first into dihydrofolate and then into tetrahydrofolate (THF), a universal one-carbon unit acceptor. DHFR is also an active enzyme in the BH4 salvage pathway, where it catalyzes the reduction of BH2 into BH4 [74]. THF accepts one-carbon units derived from the amino acids, serine and glycine, and the resulting methylated-THF exists in several interchangeable forms with varying chemical structures. These include formyl-THF, methyl-THF, and methylene-THF, which, respectively, donate their one-carbon units to purine synthesis, the methionine recycling pathway (via homocysteine methylation), and thymidylate synthesis [142].

Several studies have associated folate metabolism with increased DNA methylation in blood cells [143], liver [144], kidney [145], and gut [146], as well as, with increased concentrations of SAM in erythrocytes [147], a key metabolite involved in DNA methylation. On the other hand, it has been shown that the reduction of DHFR activity diminishes the cellular THF pool, altering the folate-dependent enzyme activity, and therefore, epigenetic profiles [148]. Folate deficits have been extensively associated with an increased risk of cardiovascular diseases, multiple cancers, and neural-tube defects due to deficient DNA methylation [149,150,151,152]. In addition, BH4 non-hereditable or genetic deficiencies have also been related to conditions where folate metabolism was reported to be compromised, including brain-maturation defects [153] and cardiovascular diseases [154]. Moreover, DHFR inhibition is essential to the action of antifolate medications used to treat cancer and some inflammatory diseases, and it is well described that methotrexate reduces BH4 levels [155], denoting the intricate association between these two metabolic pathways in regulating DNA methylation.

The practice of physical exercise is known to modulate DNA methylation, favoring the hypermethylation of some DNA regions and the hypomethylation of others. The outcome is the permissive gene expression of genes beneficial for cell health, *i.e.*, anti-inflammatory and antioxidant genes. In this scenario, it has been shown that acute resistance exercise used to stimulate hypertrophy can induce different epigenetic modifications in human skeletal muscle, including the hypermethylation of *GPAM* and *SREBF2* genes [156]. *GPAM* and *SREBF2* encode for enzymes involved in the biosynthesis of lipids, a metabolism that has been proposed to be dependent on appropriate intracellular levels of BH4 [157].

The practice of light-intensity physical activity by a general cohort of a healthy middle-aged population generated hypermethylation of the gene speck-like protein containing a caspase recruitment domain (*ASC*) in peripheral blood mononuclear cells, resulting in a decrease in expression [158]. ASC encodes an adapter protein that is necessary for inflammasome formation and the consequent activation of pro-caspase 1 and IL1-β synthesis [159]. *ASC* hypermethylation was correlated with a decrease in the pro-inflammatory cytokines IL-6, IL-8, IL-15, and TNF-a, resulting in a decrease in systemic inflammation in middle-aged individuals [158]. Furthermore, hypermethylation of *NFκB* in peripheral blood cells has also been demonstrated after low-intensity walking exercise by elderly individuals, also demonstrating a decrease in exercise-induced systemic inflammation mediated by epigenetic mechanism [160]. This scenario suggests a possible relationship between BH4 metabolism and the effects induced by exercise on DNA methylation, favoring the antioxidant response (Figure 1); however, studies in this area have not been performed to date.

## 6. Conclusions

A vast number of studies have demonstrated the beneficial role of physical exercise in potentiating the NRF2/ARE pathway. Although the mechanisms induced by physical exercise to modulate the antioxidant system are not fully elucidated, increasing evidence indicates the involvement of the BH4 pathway and epigenetic events in the process. A better understanding of which mechanistic mediators are involved in this effect will potentially allow the development of non-pharmacological strategies, or co-adjuvant therapies, that seek the prevention of chronic or neurodegenerative diseases where oxidative stress, inflammation, and mitochondrial dysfunction are involved in physiopathology.

## Figures and Tables

**Table 1:** The erythroid-related nuclear factor 2 (NRF2), antioxidant-responsive-elements (ARE) system, and enhanced enzyme activities in the brain and skeletal muscle.

Antioxidant/Detoxifying Enzyme	Reported Effect	
	Brain	Skeletal Muscle
Superoxide dismutase	↑ Resistance to neurotoxicity [19] ↓ Level of ischemic damage [20] ↓ Motoneuron degeneration [21]	↑ Protection against multiple organ dysfunction [22] ↑ Protection against diabetic cardiomyopathy [23]
Glutathione peroxidase	↑ Protection against stroke damage [24]	↑ Muscle damage recovery
Glutathione reductase	↓ Anxiety-like behavior [25]	↑ Lean mass and muscle strength [26]
Hemeoxygenase-1	↑ Protection against heat-induced brain damage [27] ↑ Improvement of ischemic injury during acute stroke [28]	↓ Sepsis-induced skeletal muscle atrophy [29] ↓ Muscle damage in Duchenne muscular dystrophy [30]
Peroxiredoxin	↑ Memory performance [31]	↑ Eccentric contraction-induced force [32]
Thioredoxin	↑ Ameliorate ischemic brain damage [33]	↑ Preservation of mitochondrial redox status [34] ↓ Muscle atrophy [35]
Metallothionein	↑ Brain aging [36] ↑ Neuroprotection after stroke [37]	↑ Regeneration in conditions of muscle wasting [38]
NAD(P)H: quinone oxidoreductase	↓ ROS and ↑cell proliferation of glioblastoma multiforme in vitro [39]	↑ Muscle degradation upon aging [40]
Glutamate cysteine ligase	↑ Learning performance [41]	↓ Susceptibility to oxidative damage in muscle aging [42]

**Table 2:** Activation of the synthesis of tetrahydrobiopterin (BH4) induced by exercise.

Physical Exercise	Population and Duration of Exercise	Sample	Neopterin and BH4 Synthesis	References
Ergometer	Normal volunteers consist of young subjects (15 to 29 y) and middle-aged subjects (40 to 59 y) undergoing strong exercise (80% VO_2max_) for 10 min	Plasma	BH4 increased by up to 150% after exercise when compared to pre-training, then rapidly returned to basal levels after 30 min	[85]
Ergometer	Normal volunteers undergoing strong exercise (80% VO_2max_) for 10 min	Plasma	BH4 increased after strong exercise and decreased after 2 h	[86]
Running	Well-trained runners covering a distance of 20 km within 2 h	Plasma	Neopterin increased 1 h after exercise for 24 h	[87]
Cycle ergometer	Healthy adults—continuous progression protocol	Plasma	Neopterin increased post-exercise and returned to basal values after 60 min	[88]
Ergometer	Healthy and trained athletes performed a 20 min maximal pedaling	Plasma	Neopterin increased post-exercise	[89]
Ultra-endurance Multi-Sport Brazil race	Well-trained male athletes undergoing 90 km alternating exercise of off-road running, mountain biking, and canoeing	Plasma	Neopterin increased post-exercise	[90]
Running	An athlete competing in the Race Across America	Urine	Neopterin increased right after the race started until day four	[91]
Rugby	Rugby match	Urine	Neopterin increased post-match and 17 h later returned to basal levels	[92]
Bodybuilding	Competitive bodybuilders who trained for 5 d in a row and 2 d off and healthy controls	Urine	Neopterin was elevated over 1 week	[93]
Triathlon	Athletes during competition	Urine	Neopterin increased post-competition	[94]
Extreme mountain ultra-marathon	Ultra-marathon runners	Urine	Neopterin increased post-race	[95]

**Table 3:** Effects of exercise-induced lncRNA modulation.

Physical Exercise	LncRNA	Reported Effect	References
Swimming	CPhar	Prevention of myocardial ischemia-reperfusion injury and cardiac dysfunction	[132]
Swimming	Mhrt779	Heart antihypertrophic effect	[133]
Treadmill	MSTRG.2625 MSTRG.1557 MSTRG.691 MSTRG.7497	Promotion of osteogenic differentiation	[134]
Treadmill	CYTOR	Regulation of fast-twitch myogenesis in aging	[135]
Aerobic exercise (single jump rope, double jump rope, round-trip running, and gymnastics)	MALAT1	Improvement of endothelial dysfunction	[136]
Swimming	LOC102633466 LOC102637865 LOC102638670	Improved motor performance	[137]
Treadmill	TUG1	Reduction of hippocampal neuronal apoptosis	[138]
Treadmill	Neat1 Meg3 Malat1 Kcnq1ot1	Possible involvement in insulin resistance and glucose homeostasis pathways	[139]
Running wheels	SNHG14	Improvement of cognitive disorder and inflammation	[140]
